# The Role of Erbium–YAG in Treating Male Genital Warts

**DOI:** 10.3390/jcm14051575

**Published:** 2025-02-26

**Authors:** Badea Jiryis, Khozayma Khamaysi, Emily Avitan-Hersh, Jonathan Shapiro, Marwan Dawood, Majd Shehadeh, Ziad Khamaysi

**Affiliations:** 1 Department of Dermatology, Rambam Health Care Campus, Haifa 3109601, Israel; badi.jiryis@campus.technion.ac.il (B.J.); e_avitan@rambam.health.gov.il (E.A.-H.); logitec_3rd@hotmail.com (M.D.); 2 Bruce Rappaport Faculty of Medicine, Technion Institute of Technology, Haifa 3200003, Israel; mshehade1@gmail.com; 3 The Azrieli Faculty of Medicine, Bar-Ilan University, Safed 1311502, Israel; zakhozaymakh@clalit.org.il; 4 Clalit Medical Services, 101 Arlozorov, Tel Aviv-Yafo 6209804, Israel; 5 Maccabi Healthcare Services, Tel Aviv-Yafo 6816330, Israel; jonmidi@gmail.com

**Keywords:** laser, human papilloma virus, condyloma acuminatum, genital warts, Er:YAG

## Abstract

**Background/Objective**: Condyloma acuminatum, also known as genital warts, results from infections of the basal epithelium or mucous membranes by human papillomavirus (HPV). These lesions can significantly impact patients’ quality of life. Recent advances in laser technology, particularly ablative lasers such as CO_2_ and Erbium–YAG (Er:YAG), have introduced new treatment opportunities. The Er:YAG laser has gained recognition as a safe and effective treatment for viral warts. This study aimed to evaluate the efficacy of Er:YAG laser treatment of male genital warts and to identify risk factors that might influence its effectiveness. **Methods**: A retrospective chart review of 102 patients who underwent Er:YAG laser wart removal between January 2019 and April 2024 was conducted. **Results**: Of the 102 patients, 61 (60%) achieved complete response by the 12-month follow-up visit. The response rates were significantly lower when there was a high number of sessions required for complete response, long duration between wart onset and laser treatment, high number of warts treated, positive smoking status, concurrent immunosuppressed state, or active metabolic disease. **Conclusions**: The Er:YAG laser is an effective method for treating recalcitrant warts. Various factors were shown to influence its efficacy.

## 1. Introduction

Genital human papillomavirus (HPV) infection leading to genital warts, also known as condyloma acuminatum, is the most common sexually transmitted disease [1,2]. Genital warts are linked to a decline in the well-being of infected individuals, as evidenced by lower quality-of-life scores [3]. HPV is highly contagious, with a 75% chance of transmission during oral, anal, or genital sexual contact with an infected individual [4]. The virus infects the basal epithelium of the skin or mucous membranes through micro-abrasions and then integrates its DNA into the host cell genome, leading to abnormal cell proliferation and growth [5].

More than 40 genotypes of this nonenveloped double-stranded DNA virus infect the anogenital area [6,7], and these are divided into low- and high-risk virus classes according to their oncogenic potential. The high-risk subtypes, especially HPV 16, 18, 31, 33, and 35, cause virtually all cervical cancers and many cancers at other anatomical sites in both men and women. The development of these dysplasias and carcinomas usually happens after a prolonged period of infection [8,9].

Over 90% of external genital warts are caused by low-risk HPV types 6 and 11 [10]. These viruses are considered non-oncogenic and cause biologically benign proliferations, often found at sites traumatized during sexual intercourse.

Various treatment options are available for male genital warts. Chemical options include imiquimod, podophyllin, and interferons (IFNs). Physical options consist of cryotherapy, laser vaporization, and electrocautery. Other options, like cryotherapy and intralesional Bleomyicn, have also been reported. However, lesions may be refractory or recurrent to all of these treatment methods [11,12].

Laser treatments can lead to complications such as pain, discomfort, and burns. Patients may also experience erythema, blistering, or pigmentary changes, especially with improper laser use. Additionally, the Koebner phenomenon can occur, where skin trauma from the laser triggers new lesions. For instance, there have been reports of wart occurrences at laser application sites attributed to this phenomenon [13].

Laser technology has introduced novel approaches utilizing energy-based devices in the treatment of warts [14]. Different types of lasers have been used for wart treatment, including non-ablative lasers, such as pulsed-dye lasers (PDLs) or neodymium-doped yttrium aluminum garnet (Nd:YAG), and ablative lasers, such as CO_2_ and erbium-doped yttrium aluminum garnet (Er:YAG). The Er:YAG laser, operating at a wavelength of 2940 nm, falls into the category of ablative lasers similar to the CO_2_ laser. This laser is strongly absorbed by water and causes an ablation to the tissue by its photothermal effect. It has gained recognition as a secure method for addressing viral warts. Nevertheless, a considerable proportion of patients may experience a notable recurrence rate, necessitating supplementary interventions [15].

A review by Iranmanesh and colleagues [16] concluded that ablative lasers generally require fewer treatment sessions to achieve clearance compared to non-ablative lasers (PDL and Nd:YAG). Although few studies have assessed the efficacy of Er:YAG for treating male genital warts, its selectively stronger absorption by water compared to CO_2_ laser energy may be associated with fewer adverse effects [17,18]. We present the first study evaluating the safety and efficacy of the Er:YAG laser in treating male genital warts.

## 2. Materials and Methods

This retrospective study reviewed the computerized records for male patients with genital warts treated with an Er:YAG laser between January 2019 and April 2024 at the Rambam Health Care Campus. Exclusion criteria included previous laser treatments for condyloma. The study was approved by the institutional Helsinki Ethical Committee.

Laser treatments were performed using the Harmony XL Pro (Alma Lasers, Caesarea, Israel) platform, which features an Er:YAG laser, emitting light at 2940 nm. The Er:YAG laser parameters used included spot sizes of 1 or 4 mm, a frequency of 2 Hz, a fluence range of 700–2500 mJ/pulse, and a pulse duration of 1 or 2 ms. The treatment aimed to ablate the full surface of the wart with an additional 1 mm of adjacent skin. Following a 30 min topical application of a eutectic mixture of local anesthetics (EMLA cream), intralesional lidocaine was injected into each wart to minimize procedural pain and discomfort. All lesions were treated by the same physician.

Treatment sessions were scheduled every two months and continued until the condylomas were completely cleared or until 12 months after the first session. Treatment success was determined based on the complete clearance of condylomas, defined as the absence of visible and palpable lesions 2 months after the last treatment session. A 2-month interval between treatment sessions was selected to allow for adequate wound healing following the ablative laser treatment, enabling the assessment of residual or new wart development.

Patient characteristics and treatment parameters collected included the number of sessions until complete response, age, history of metabolic diseases, immunological status, smoking status, duration of condylomas before laser treatment, history of previous treatments for warts, and the number of condylomas treated.

In addition to the above parameters, this study also considered potential confounding factors that might influence treatment outcomes, such as patient adherence to follow-up visits and variations in post-treatment care. All patients received standardized post-operative instructions to minimize complications and ensure optimal healing. These instructions included guidelines on wound care, activity restrictions, and pain management. Additionally, any adverse events or complications were systematically recorded to provide a comprehensive safety profile of the Er:YAG laser treatment. By meticulously documenting these variables, this study aimed to ensure a robust analysis of both efficacy and safety outcomes.

### Statistical Analysis

Descriptive statistics, including mean, standard deviation, median, and percentage, were calculated for parameters. The normal distribution of continuous parameters was tested using the Kolmogorov–Smirnov test. Logistic regression with odds ratios and 95% confidence intervals was used to predict independent parameters influencing complete response. A *p*-value of <0.05 was considered significant. Statistical analysis was performed using SPSS version 28.

## 3. Results

Between January 2019 and April 2024, 135 male patients were treated for the first time with an Er:YAG laser for genital warts. Among them, 102 patients had their complete data collected and completed follow-up. As presented in Table 1, 61 patients (60%) demonstrated complete resolution. The mean age was 39.9 ± 14.3 years in both the complete and non-complete-response groups. The mean duration of genital warts before treatment was 4.21 months in the complete-response group and 12.9 months in the non-complete-response group. The mean number of warts per patient was 12 (range 8–20) in the complete-response group and 39 (range 24–53) in the non-complete-response group. The mean number of treatment sessions was 2.13 in the complete-response group and 4.37 in the non-complete-response group.

In the complete-response group, 8 patients (13.1%) were smokers, 0 were immunosuppressed, 17 patients (27.9%) had metabolic disease, and 51 patients (83.6%) had previous cryotherapy treatments (Table 1). In the non-complete-response group, 36 patients (87.8%) were smokers, 12 patients (29.3%) were immunosuppressed (1 patient due Hodgkin’s lymphoma and the others due to immunosuppressive drugs), 30 patients (73.2%) had metabolic disease, and 34 patients (82.9%) had previous cryotherapy treatments (Table 1). Comparing the two response groups, the statistically significant characteristics were the number of sessions required for complete response, the duration of warts before laser treatment, the number of warts treated, smoking status, immunosuppressive conditions, and the presence of metabolic disease.

**Figure 1:**
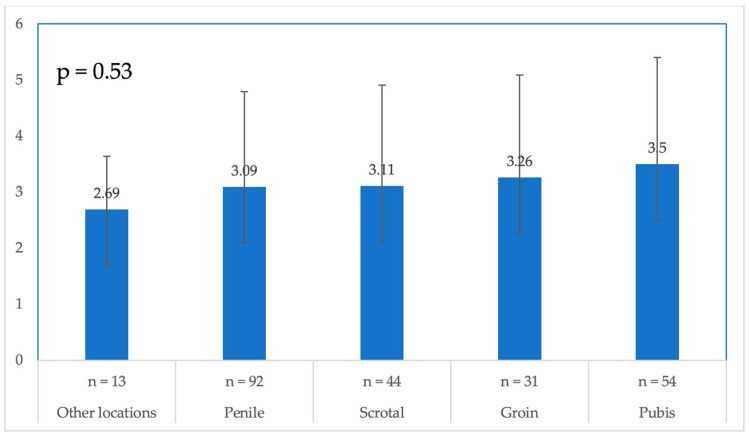
Mean number of sessions needed in each location. n = number.

Table 2 presents the number of patients with and without a complete response for each wart location. Figure 1 shows the mean number of sessions needed in each location; there was no statistically significant difference in the number of treatment sessions needed among the different wart locations, except for the pubic area, as compared to ‘other locations’, with a *p*-value of 0.03.

**Figure 2:**
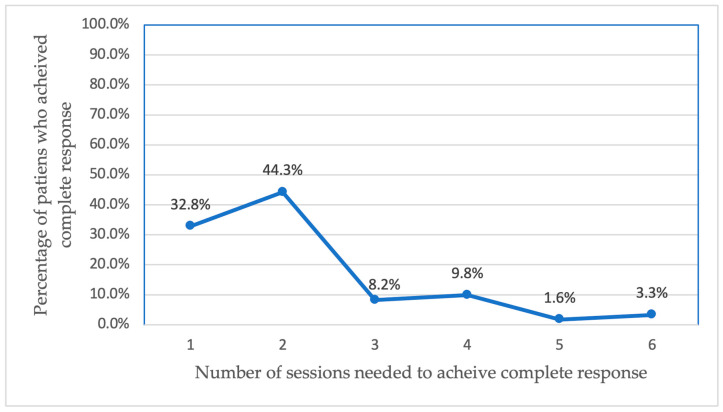
Complete response rates based on the number of sessions required.

A multivariate statistical analysis showed two significant factors affecting the response type, smoking status, with an odds ratio = 0.015 and *p*-value < 0.001, and the number of treatment sessions required to achieve a complete response, with an odds ratio = 0.317 and *p*-value < 0.001. Figure 2 illustrates that a higher number of treatment sessions was associated with a lower likelihood of achieving a complete response. Patients who achieved a complete response generally required fewer treatment sessions, while patients who did not achieve a complete response underwent more treatment sessions.

## 4. Discussion

Genital warts are sexually transmitted infections of the basal epithelium of the skin or mucous membranes that pose challenges in terms of treatment. Condyloma acuminata may recur following topical treatments; surgical excision remains the only treatment with a nearly 100% clearance rate [19,20].

Recently, laser therapy has received growing attention as an alternative treatment for male genital warts. Ablative lasers, such as Er:YAG and CO_2_ lasers, and non-ablative lasers, including long-pulsed Nd:YAG and PDL, are increasingly being used for this purpose. The mechanisms of action of non-ablative lasers differ from those of ablative lasers. PDL destroys large, dilated blood vessels within the dermal papillae [18], and the Nd:YAG laser relies on targeting dilated blood vessels through coagulation (photothermal effect) or blasting (photomechanical effect) of the target tissue, which warm and burst rapidly, with purpura formation and subsequent wart destruction [21].

Ablative lasers demonstrate higher efficacy than the non-ablative lasers [16]. They operate by emitting wavelengths that are highly absorbed by water molecules, leading to heating of the tissue up to the point of ablation and evaporation of warts, coagulation of underlying blood vessels, and necrosis of the lesion [22]. The shallow penetration depth and high power allow for precise ablation of soft tissue, like conventional scalpels, leading to fewer side effects and reduced scarring compared to CO_2_ lasers. The precision and reduced collateral damage rendered the Er:YAG laser the preferred choice for the current study [23,24].

Although Er:YAG laser therapy for genital warts has not been previously presented, the safety and efficacy of Er:YAG for other types of warts have been demonstrated. Wollina and his colleagues [15] reported a clearance rate of 72.5% in 69 patients with common warts after a single treatment session with an Er:YAG laser. A similar effective rate of 77.5% was reported by Song and his colleagues [25] when Er:YAG was combined with photodynamic therapy in treating facial flat warts, while Balevi and his colleagues [18] had a complete response rate of 83.3% in treating facial flat warts. Our previous prospective clinical trial on Er:YAG laser treatment for warts reported a complete resolution rate ranging from 29% (Er:YAG monotherapy) to 48% (combined Er:YAG + LP Nd:YAG), with the combined approach showing statistically superior results (*p* = 0.008) [26]. More recently, we presented a retrospective study in which a 71.6% complete resolution rate was observed at 12 months with Er:YAG monotherapy [27].

The clinical decision to use an Er:YAG laser over other treatment modalities is influenced by its precision and minimal thermal damage to surrounding tissues. Additionally, the ability to adjust parameters such as spot size, fluence, and pulse duration allows for tailored treatments to individual patient needs. This flexibility enhances patient comfort and reduces recovery time, making it a preferred choice for sensitive areas such as the genital region. Understanding these advantages provides a comprehensive perspective on the clinical utility of Er:YAG laser therapy in treating genital warts.

Our current analysis found that 61 out of 102 patients achieved a complete response within 12 months of treatment, yielding a success rate of 60% for Er:YAG laser treatment. An inverse relationship between the number of treatment sessions and the complete response rate was noted, suggesting that patients who do not achieve a complete response in early sessions may face challenges later. Wart location also significantly impacted treatment outcomes, with pubic area warts showing a reduced likelihood of complete response. Healthcare providers should factor this into their treatment plans, as pubic warts may require more intensive or ^21^combination therapy. This also underscores the need for patient education and avoiding shaving this area, which could exacerbate the lesions.

Smoking also appeared to influence treatment response, with 87.8% of non-responders being smokers compared to only 13.1% of responders who smoked. The statistical analysis demonstrated an odds ratio of 0.015, indicating that smoking significantly reduces the likelihood of achieving a complete response.

The analysis also demonstrated that the number of warts treated impacted the response, with fewer warts correlating with better outcomes. Similarly, a shorter duration of wart onset to laser treatment was associated with better results, suggesting that early intervention is advantageous. Other factors negatively affecting treatment success included a history of immunosuppression or metabolic disease. In contrast, factors such as patient age, ethnicity, and previous wart treatments did not influence laser treatment outcomes.

Researchers and healthcare professionals should further investigate the effectiveness of laser treatments and assess the impact of variables such as treatment intensity, patient tolerance, and suitability on treatment outcomes. A deeper understanding of these factors would improve treatment precision.

## 5. Conclusions

The Er:YAG laser is an effective treatment for male genital warts, with the best results observed in patients who were non-smokers, non-immunosuppressed, and without current metabolic diseases, with warts of a short duration located outside of the pubic area. Patients who do not achieve complete resolution in initial sessions may benefit from further combination therapy.

## Figures and Tables

**Table 1:** Patient characteristics.

Patient Characteristic	No Complete Response N = 41 ^a^	Complete Response N = 61 ^a^	*p*-Value
Age	39.9 ± 10.8	40 ± 14.3	*p* = 0.99
Number of sessions	4.37 ± 1.5	2.13 ± 1.2	*p* < 0.001
Onset until laser treatment (months)	12.9 ± 7.4	4.21 ± 1.4	*p* < 0.001
Number of warts treated mean	39 [24–53]	12 [8–20]	*p* < 0.001
Smoker	36 (87.8)	8 (13.1)	*p* < 0.001
Immunosuppressed	12 (29.3)	0	*p* < 0.001
Metabolic disease	30 (73.2)	17 (27.9)	*p* < 0.001
Previous topical treatment	38 (92.7)	51 (83.6)	*p* = 0.23
Previous cryotherapy treatment	34 (82.9)	51 (83.6)	*p* = 0.93

N = number of patients. ^a^ N (%) or mean +/− standard deviation [range].

**Table 2:** Number of responders and non-responders according to anatomical location of the warts.

Wart Site	No Complete Response N = 41 ^a^	Complete Response N = 61 ^a^	*p*-Value
Groin	15 (48.4)	16 (51.6)	*p* = 0.27
Pubis	33 (61.1)	21 (38.9)	*p* < 0.001
Scrotal	20 (45.5)	24 (54.5)	*p* = 0.38
Penile	38 (41.3)	54 (58.7)	*p* = 0.74
Other location	2 (15.4)	11 (84.6)	*p* = 0.069

N = number of patients. ^a^ N (%) or mean +/− standard deviation [range].

## Data Availability

The data presented in this study are available on request from the corresponding author due to legal and ethical reasons.

## Abbreviations

The following abbreviations are used in this manuscript:

Er:YAG	Erbium-doped yttrium aluminium garnet
Nd:YAG	Neodymium-doped yttrium aluminum garnet
PDL	Pulsed-dye
HPV	Human papillomavirus
